# Prey tracking and predator avoidance in a Neotropical moist forest: a camera-trapping approach

**DOI:** 10.1093/jmammal/gyac091

**Published:** 2022-11-11

**Authors:** Constant Swinkels, Jessica E M van der Wal, Christina Stinn, Claudio M Monteza-Moreno, Patrick A Jansen

**Affiliations:** Wildlife Ecology & Conservation, Department of Environmental Sciences, Wageningen University, 6708 PB Wageningen, The Netherlands; Smithsonian Tropical Research Institute, Balboa, Ancón 0843-03092, Republic of Panama; Plant Ecology and Physiology, Radboud University, 6525 AW Nijmegen, The Netherlands; FitzPatrick Institute of African Ornithology, University of Cape Town, Cape Town 7701, South Africa; Smithsonian Tropical Research Institute, Balboa, Ancón 0843-03092, Republic of Panama; Department of Conservation Biology, Georg-August-Universität Göttingen, 37073 Göttingen, Germany; Smithsonian Tropical Research Institute, Balboa, Ancón 0843-03092, Republic of Panama; Department for the Ecology of Animal Societies, Max Planck Institute of Animal Behavior, 78315 Konstanz, Germany; Wildlife Ecology & Conservation, Department of Environmental Sciences, Wageningen University, 6708 PB Wageningen, The Netherlands; Smithsonian Tropical Research Institute, Balboa, Ancón 0843-03092, Republic of Panama

**Keywords:** Barro Colorado Island, camera trapping, Central American agouti, ocelot, predator avoidance, prey tracking, survival analysis, temporal proximity logging

## Abstract

Whether prey species avoid predators and predator species track prey is a poorly understood aspect of predator–prey interactions, given measuring prey tracking by predators and predator avoidance by prey is challenging. A common approach to study these interactions among mammals in field situations is to monitor the spatial proximity of animals at fixed times, using GPS tags fitted to individuals. However, this method is invasive and only allows tracking of a subset of individuals. Here, we use an alternative, noninvasive camera-trapping approach to monitor temporal proximity of predator and prey animals. We deployed camera traps at fixed locations on Barro Colorado Island, Panama, where the ocelot (*Leopardus pardalis*) is the principal mammalian predator, and tested two hypotheses: (1) prey animals avoid ocelots; and (2) ocelots track prey. We quantified temporal proximity of predators and prey by fitting parametric survival models to the time intervals between subsequent prey and predator captures by camera traps, and then compared the observed intervals to random permutations that retained the spatiotemporal distribution of animal activity. We found that time until a prey animal appeared at a location was significantly longer than expected by chance if an ocelot had passed, and that the time until an ocelot appeared at a location was significantly shorter than expected by chance after prey passage. These findings are indirect evidence for both predator avoidance and prey tracking in this system. Our results show that predator avoidance and prey tracking influence predator and prey distribution over time in a field setting. Moreover, this study demonstrates that camera trapping is a viable and noninvasive alternative to GPS tracking for studying certain predator–prey interactions.

## Abstract (es)

Debido a las dificultades en evaluar como los depredadores rastrean su presa, y como las presas eluden a depredadores, un aspecto todavía poco conocido en la interacción entre depredadores y presas es si estas evitan a depredadores o si estos rastrean las presas. Una enfoque común para estudiar estas interacciones entre mamíferos bajo condiciones de campo es de seguir la proximidad espacial de animales a intervalos fijos, usando marbetes de GPS ajustados a los individuos. Sin embargo, este método es invasivo y solo permite obtener información de un número limitado de individuos. En este estudio, usamos cámaras trampas como método alternativo y no invasivo, para monitorear el proximidad temporal entre depredadores y presas en sitios fijos en Isla Barro Colorado, Panamá, donde los ocelotes (*Leopardus pardalis*) son el principal mamífero depredador. Evaluamos dos hipótesis: (1) las presas evitan a los ocelotes, y (2) los ocelotes rastrean a las presas. Cuantificamos la proximidad temporal de depredadores y presas ajustando modelos paramétricos de supervivencia a los intervalos de tiempo ocurridos entre observaciones subsecuentes en las cámaras trampas, y luego comparamos los intervalos observados con permutaciones aleatorias que retuvieron la distribución espacio-temporal de la actividad de los animales. Encontramos que, si un ocelote había pasado por dicha área, el tiempo en el cual una presa aparece en una ubicación fue significativamente mayor que lo esperado aleatoriamente. También encontramos que, después de pasar una presa por un área, el tiempo en que un ocelote tarda en aparecer fue significativamente menor que lo esperado al azar. Estos resultados constituyen evidencia indirecta que las presas evitan a los depredadores y que estos rastrean a las presas. Nuestros resultados muestran que la evitación de los depredadores y el rastreo de las presas influyen en la distribución de presas y depredadores a lo largo del tiempo en un escenario natural. Este estudio también demuestra que las cámaras trampas son una alternativa viable y no invasiva, con respecto a marbetes de GPS, para estudiar ciertas interacciones entre presas y depredadores.

Predator–prey dynamics are central to shaping ecosystems (MacArthur 1955; Hairston et al. 1960; Paine 1966; Crooks and Soulé 1999). The interactions between predators and their prey have important implications for the structure and function of ecological communities (e.g., Schmitz et al. 1997; Brose et al. 2019; Sommers and Chesson 2019). Predators not only suppress populations of prey species but also affect the spatial distribution of prey animals, thereby influencing landscape-wide patterns of feeding and consequently vegetation development (e.g., Gude et al. 2006; Frank 2008). Understanding how predator–prey dynamics drive the spatial distribution and density of animals at the local and landscape level is therefore important information for nature restoration and conservation projects.

Many studies have tried to understand whether and how prey avoid predators (i.e., predator avoidance) and whether and how predators track prey (i.e., prey tracking), and how such interactions impact the spatiotemporal behavior of predators and prey (e.g., Lima and Dill 1990; Lima 1998; Brown and Kotler 2004). The “landscape of fear” hypothesis, for example, states that prey respond to spatial variation in predation risk, resulting from predators concentrating in areas with high hunting success (Laundré et al. 2001; Brown and Kotler 2004; Creel and Winnie 2005; Grant et al. 2005). Under this hypothesis, prey species would try to avoid high-risk areas while simultaneously taking risks to gather sufficient resources (Berger-Tal and Bar-David 2015; Gallagher et al. 2017).

Mapping fine-scale predator–prey dynamics is imperative to understanding and ultimately predicting how predator–prey interactions can influence species coexistence and species distribution—information that is valuable for nature conservation planning. Controlled experiments have shown that prey try to avoid places where predators have recently been active (Sündermann et al. 2008; Ferrero et al. 2011), and conversely that predators are attracted to places where prey have recently been active (Hughes et al. 2010). However, detailed insights into fine-scale predator avoidance and prey-tracking mechanisms in real-world settings are challenged by the difficulty of tracking small-scale movements of freely moving predators and their prey (Maffei et al., 2005; Emsens et al., 2014).

A common Lagrangian approach, where one specific object is followed through space and time, to measure fine-scale predator–prey dynamics in the field involves measuring the spatial proximity of predators and prey at fixed times by simultaneously logging the positions of both, using GPS tags mounted on individuals (e.g., Kranstauber et al. 2017; Schmitz et al. 2017). Examples are studies that have measured (both short- and long-term) avoidance responses of prey to predator proximity (e.g., Latombe et al. 2014; Basille et al. 2015). However, this method is invasive, often costly, and subsequently typically only allows a subset of the population to be followed simultaneously. Given that many untagged individuals remain invisible, it is not possible to observe the majority of interactions and, as such, results should be interpreted with caution (Creel et al. 2013).

An alternative Eulerian approach, where flow of objects through time on one specific point in space is observed, is to measure the temporal proximity of predators and prey at fixed locations using camera traps (Smith et al. 2020), which monitor the visitation of a small habitat patch by animals. Camera traps have already been extensively used to measure activity patterns and spatial distributions as predator avoidance mechanisms by comparing population-level patterns of daily activity and habitat use between predators and prey (e.g., Harmsen et al. 2009; Wang and Fisher 2012; Suselbeek et al. 2014; Rota et al. 2016). To more directly map prey tracking or predator avoidance patterns, we can analyze time intervals between animal visits. Several studies have used this approach in man-altered environments and experimental settings (e.g., Ford and Clevenger 2010; López-Bao et al. 2011; Cusack et al. 2017; Moll et al. 2018; Martinig et al. 2020; Randler and Kalb 2020), but whether and how predator–prey dynamics influence species distribution and behavior in natural systems remains poorly understood.

In this study we analyzed time intervals between prey and predators from camera traps to determine whether dynamic interactions between prey and predators occur in a complex and biodiverse ecosystem. We did this on Barro Colorado Island (BCI), Panama, where the ocelot (*Leopardus pardalis*) is the principal mammalian predator hunting mostly rodents and birds. We hypothesized that (1) prey avoid ocelots, and (2) ocelots track prey. We tested two corresponding predictions: (i) prey take longer than may be expected by chance to appear in places that an ocelot has visited; and (ii) predators appear sooner than may be expected by chance to appear in areas that have been visited by prey.

## Materials and Methods

### Site and species

**Fig. 1.**
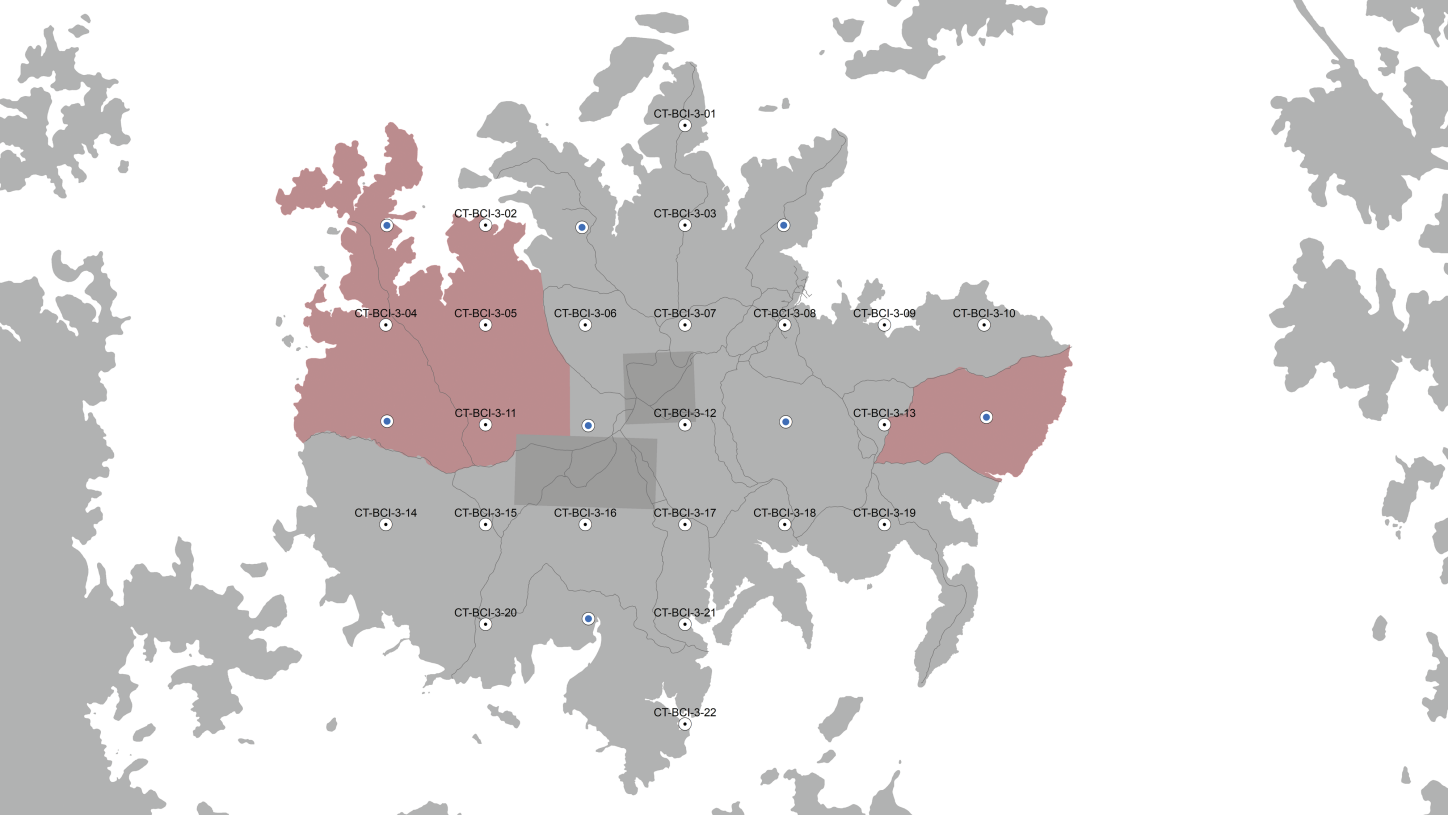
—Map of Barro Colorado Island showing the locations at which camera traps monitored the mammal community. Lines indicate the trail system. Research-free areas are shaded red. Filled dots are points shared with the grid of the Tropical Ecology Assessment and Monitoring (TEAM) program (Jansen et al. 2014). The dark gray squares represent permanent vegetation survey plots.

Camera traps were deployed across BCI in Panama (9°10ʹN, 79°51ʹW; Fig. 1), an island of 15.6 km^2^ in the Gatún Lake section of the Panama Canal covered by semideciduous lowland moist tropical forest. Annual rainfall averages 2,600 mm, with a notable dry period between late December and April (Leigh 1999). BCI has been protected from poaching since 1960 and exhibits a rich mammal fauna as a result, but nowadays lacks permanent presence of jaguar and puma, the top predators (Wright et al. 1994).

The most common mammalian predator on BCI is the ocelot, a felid that is largely nocturnal but can also be active during the day (Aliaga-Rossel et al. 2006; Moreno et al. 2006, 2012; Emsens et al. 2014; Suselbeek et al. 2014). Home ranges and travel distances of the ocelot on BCI differ between males (respectively, 3.48 km^2^ and 1.15 km) and females (respectively, 1.48 km^2^ and 0.7 km) (Moreno et al. 2012). Ocelots hunt on the ground. Their diet in Panama consists mostly of around 50 medium-sized prey species (Sunquist and Sunquist 2002). On BCI, the Central American agouti (*Dasyprocta punctata*) makes up most of its diet, about 60% (Moreno et al. 2006). Other prey animals of the ocelot include the great tinamou (*Tinamus major*), juvenile collared peccary (*Pecari tajacu*), paca (*Cuniculus paca*), white-nosed coati (*Nasua narica*), common opossum (*Didelphis marsupialis*), gray-chested dove (*Leptotila c. cassinii*), spiny rat (*Proechimys semispinosus*), and nine-banded long-nosed armadillo (*Dasypus novemcinctus*; Pratas-Santiago et al. 2016). For a complete account of the species on BCI, see https://stricollections.org/portal/checklists.

The principal prey species of the ocelot, the Central American agouti, is also the most common terrestrial mammal of BCI. This rodent occurs from southern Mexico to northern Colombia, has a home range of about 2–4 ha, and weighs 2–4 kg. Refugia include burrows, logs, and dense vines (Smythe 1978; Aliaga-Rossel et al. 2008; Emsens et al. 2013). The agouti is primarily diurnal with occasional nocturnal activity (Lambert et al. 2009). The diet consists mainly of large seeds such as those of the palm species *Astrocaryum standleyanum* (Smythe 1978, 1989; Hirsch et al. 2012; Jansen et al. 2012; Emsens et al. 2013). On BCI, the main cause of death for agouti is predation by ocelots (Aliaga-Rossel et al. 2006; Suselbeek et al. 2014).

### Data collection and processing

Data were collected with unbaited, motion-triggered camera traps (PC900; Reconyx Inc., Holman, Wisconsin) operated between 2015 and 2017. The cameras were placed in a 30-point grid across the island with 0.7-km interspacing (Fig. 1; camera density of 2 km^−2^) without prior knowledge of the field situation at each grid point. Cameras were mounted at 50 cm from the ground on a tree close to the grid point and were not aimed at a trail or other particular feature but with a clear line of sight. Cameras recorded for up to 3 months at a time and were replaced 4–6 times per year. Cameras were programmed to take 10 time-stamped photographs upon every movement trigger of a passive infrared sensor, and to take one time-lapse photograph every 12 h. The time-lapse photograph allowed us to approximate the time at which a camera stopped working in cases where it failed before the next pickup/setup moment. We used this final time-lapse photograph before battery failure to calculate the time between the last animal passing and camera failure. This interval was then analyzed as a censored event (see “Statistical analysis”). We identified animals to the species level and archived data using the application “Agouti” (Casaer et al. 2019).

We omitted all false observations (*n* = 2,527; e.g., plants moving in the breeze) and all triggers from species not known to be prey of ocelot and unlikely to interact with ocelot (*n* = 185; a list of all omitted observations can be found in Supplementary Data SD1). We included humans (*Homo sapiens*, *n* = 34), jaguarundi (*Herpailurus yagouaroundi*, *n* = 2), crab-eating raccoon (*Procyon cancrivorus*, *n* = 8), and tayra (*Eira barbara*, *n* = 11) in our analysis as “nontarget animals” that potentially disrupted interactions between ocelot and their prey (see “Statistical analysis”). The remaining triggers were from predators (ocelot) and their prey, from which we calculated interval times between consecutive observations and classified intervals based on the identity (prey or predator) of the animals. We omitted from the analyses all time intervals longer than 3 days (*n* = 421), given that animals passing a camera more than 3 days apart are unlikely to be interacting.

### Statistical analysis

We performed all analyses in R 3.4.3 (R Core Team 2017). To verify that prey and predator activity patterns were sufficiently overlapping, we calculated daily activity patterns (kernel density, 250 reps) of ocelot and its prey species at BCI using the package “activity” (Rowcliffe et al. 2014). To test whether we could measure predator avoidance and prey tracking from time intervals in a real-world setting, we applied parametric survival analyses. Survival analyses allow analysis of time-to-event data and allow incorporation of “censored” data in which the event is not observed. To assess predator avoidance, we analyzed the time interval between the passing of a predator (ocelot) and a consecutive prey (the event of interest). To assess prey tracking, we analyzed the time interval between the passing of a prey and a consecutive predator (the event of interest). We included two types of censored data: (i) incomplete intervals due to camera failure or pickup; and (ii) intervals that were interrupted by a nontarget prey or predators (known as “competing events”; Heisey and Patterson 2006).

To test the null hypotheses that temporal proximity of predators and prey was random given the activity patterns of the species, we compared our observed data to 999 random distributions (generated using the package “permute”; Simpson et al. 2019). Specifically, within each location, we shuffled the date among observations to retain the spatial pattern in local and seasonal abundance of animals across the data set, while keeping time and species together to retain the activity patterns of specific species (see Supplementary Data SD2 for the visualized workflow). For each run, we fitted a parametric survival model with a Weibull distribution and calculated the constant hazard rate using the “survreg” function in the package “survival” (Therneau et al. 2022). The constant hazard rate provides a convenient single value that describes the distribution of interval times over the survival curve. In addition, we report the time at which 50% of the intervals had expired (median time) to facilitate interpretation. We then calculated the significance of the observed constant hazard rate as the percentage of these 999 randomly generated constant hazards that exceeded the observed hazard rate (significance at alpha = 0.05). We plotted the survival probabilities over time using the package “survminer” (Kassambara et al. 2021).

We ran the analysis using three alternative approaches to determine whether decisions on data handling influenced the results. First, we used a 7-day interval cutoff (omitting 103 intervals) instead of the 3-day cutoff, as interaction of predators with prey scent trails has been described after such a time period (Koivula and Korpimäki 2001). Second, we reran the analysis on prey tracking (prey–predator events) without competing events, as inclusion of prey–prey competing events in the analysis of prey–ocelot events resulted in a large number of competing events and high uncertainty in the survival curve, because prey (*n* = 34,357) were 100× more common than predators (*n* = 295). We present this approach, as it is more intuitive. Finally, we included humans, jaguarundis, tayras, and crab-eating racoons as predators (events of interest) instead of as nontarget animals (censors), as they could potentially invoke a similar response in prey animals as ocelots.

## Results

**Fig. 2.**
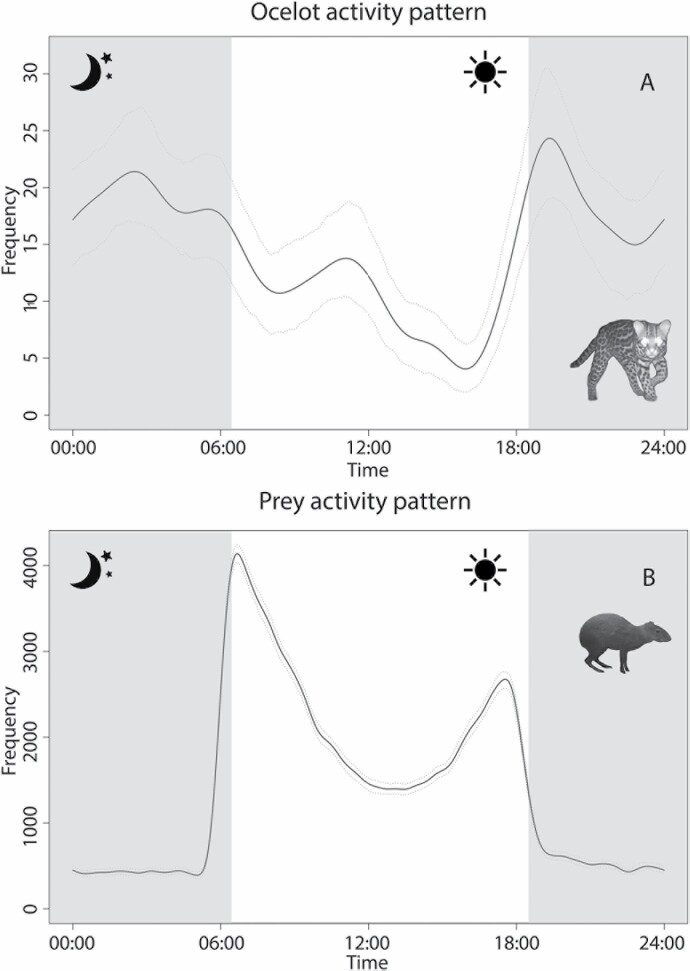
—Daily activity patterns (kernel density, 250 reps) of ocelot (A) and its prey species (B) at Barro Colorado Island, Panama. Curves and 95% confidence envelopes were fitted to time stamps of camera-trap records. Images of ocelot and agouti were made by Helen J. Esser.

Our analysis was based on 35,194 observations, including 295 observations of ocelots, 55 of potential nontarget predators, 34,357 of prey (Table 1), and 487 final time-lapse photographs and setup/pickup triggers used to approximate the time at which a camera stopped working. Interval times followed a Weibull distribution (median = 2.80 h, mean ± *SE* = 8.15 h ± 0.08 h). Ocelot activity was distributed across the entire day, although biased to the night (Fig. 2A). Prey activity was predominantly diurnal (Fig. 2B). The daily activity pattern of prey resembled that of the dominant species, the Central American agouti. Thus, there was substantial overlap in daily activity patterns between ocelot and their prey.

**Table 1.** —List of predator and prey species recorded by a grid of camera traps on Barro Colorado Island, Panama, with the number of observations, the number of locations (out of 30), and the capture rate (average number of observations per week).

Species	Scientific names	Number of observations	Number of locations	Capture rate (week^−1^)
Predators				
Ocelot	*Leopardus pardalis*	295	30	0.193
Human*	*Homo sapiens*	34	30	0.022
Tayra*	*Eira barbara*	11	9	0.007
Crab-eating racoon*	*Procyon cancrivorus*	8	6	0.005
Jaguarundi*	*Herpailurus yagouaroundi*	2	2	0.001
Prey				
Agouti	*Dasyprocta punctata*	22,532	30	14.76
Collared peccary	*Pecari tajacu*	4,911	30	3.216
Paca	*Cuniculus paca*	2,824	30	1.849
Red brocket deer	*Mazama americana*	1,905	30	1.248
White-nosed coati	*Nasua narica*	1,147	30	0.751
Red-tailed squirrel	*Sciurus granatensis*	217	24	0.142
Great tinamou	*Tinamus major*	213	25	0.139
Common opossum	*Didelphis marsupialis*	180	23	0.118
Gray-chested dove	*Leptotila c. cassinii*	146	15	0.096
Nine-banded long-nosed armadillo	*Dasypus novemcinctus*	90	13	0.059
Spiny rat	*Proechimys semispinosus*	76	17	0.050
White-faced capuchin	*Cebus imitator*	60	19	0.039
Northern tamandua	*Tamandua mexicana*	45	15	0.029
Green iguana	*Iguana iguana*	8	2	0.005
Brown four-eyed opossum	*Metachirus nudicaudatus*	1	1	0.001
Robinson’s mouse opossum	*Marmosa robinsoni*	1	1	0.001
Howler monkey	*Alouatta palliata*	1	1	0.001

*Species included as competing events.

### Prey tracking by predators

**Fig. 3.**
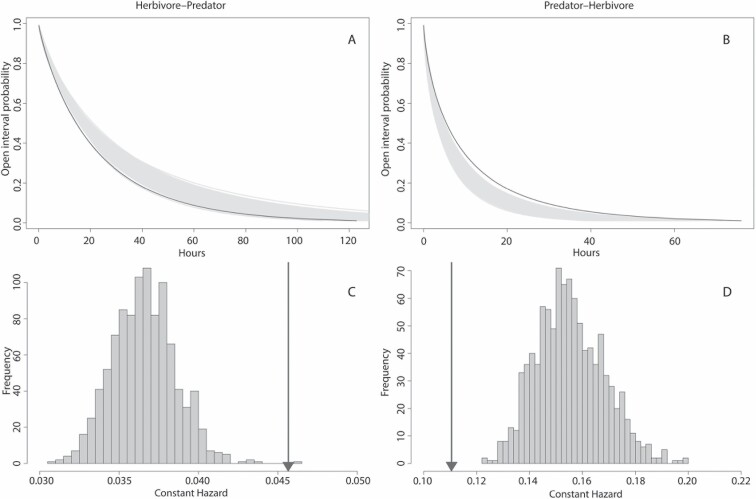
—Evidence for prey tracking by ocelots (A, C) and ocelot avoidance by prey (B, D) on Barro Colorado Island, Panama. Survival probability curves (A, B), fitted using a Weibull distribution, quantify respectively ocelot–prey (predator avoidance) and prey–ocelot (prey tracking) intervals recorded by camera traps. The gray lines represent 999 random distributions, and the black line is the observed distribution. Distributions of the constant hazard rate (C, D) as derived from the random survival probability curves in A and B, respectively (gray bars). The arrows indicate the observed hazard rate.

On average, ocelots appeared sooner after an observed prey animal than would be expected from null distributions where animals randomly pass by, visible in Fig. 3A by the black line outside the distribution of randomized distributions in gray. The median time interval was 14.83 h for the observations and 17.86 h ± 0.03 h for the null distribution. The observed hazard rate was higher than 99% of all values generated by 999 randomizations, hence significant (Fig. 3C). The difference was significant regardless of whether competing events resulting from consecutive prey events were excluded (Fig. 3C; *P* < 0.01), or not (Supplementary Data SD3; *P* < 0.001). Using the alternative 7-day cutoff or including humans and other potential predators as target events did not significantly change the outcomes (Supplementary Data SD4C–F and SD5C–F). These results are consistent with prey tracking by ocelots.

### Predator avoidance by prey

On average, prey appeared significantly later after the passage of an ocelot than would be expected by chance, visible in Fig. 3B by the black line outside the randomized distribution. The median time interval was 5.43 h for the observations and 3.92 h ± 0.01 h for the null distribution. The observed constant hazard rate was smaller than all thousand constant hazard rates for randomized intervals (Fig. 3D; *P* < 0.001). Using the alternative 7-day interval cutoff and including humans and other predators as target events did not significantly change the outcomes (see Supplementary Data SD4A, B and SD5A, B). This result is consistent with predator avoidance.

## Discussion

We present empirical evidence for predator avoidance by prey, and prey tracking by predators in a Neotropical mammal community. To the best of our knowledge, this is the first study that directly measures predator avoidance and prey tracking in a field setting including the complete animal community, and also the first that analyzes intervals from camera-trapping data with survival analyses and random permutations. Our study helps to improve our understanding of predator–prey dynamics, which are central to shaping ecosystems.

We found that time intervals between ocelot and prey visits to camera-monitored locations were significantly longer than expected by chance. This indicates that prey avoided places that had been recently visited by ocelots, in line with the hypothesis of predator avoidance by prey. This finding implies that predator avoidance as observed in controlled experiments (Sündermann et al. 2008; Ferrero et al. 2011) also occurs in a field setting. It adds to field studies that showed that GPS-collared prey avoided locations where predators had recently hunted (Liley and Creel 2008; Latombe et al. 2014).

Ocelot visits to camera-monitored locations occurred significantly sooner after visits of prey than expected by chance, which is in line with the hypothesis of prey tracking by predators. This finding adds evidence from a field situation to prior experiments. Hughes et al. (2010), for example, found that free-roaming predators were drawn to places to which mouse scent had been experimentally applied. Likewise, Emsens et al. (2014) found that ocelots actively sought out refuges of their principal prey, the Central American agouti, on BCI.

Our findings suggest that ocelots and their prey in our study system are engaged in a dynamic game in which the prey animals show fine-scale avoidance of locations once their predator has visited it. Although predators were primarily active at night and prey primarily during the day, activity patterns in our study did overlap (also see Suselbeek et al. 2014), suggesting this game affects the entire prey community throughout the day and night. It is easy to imagine that repeated use of locations by predators—for example, due to proximity of dens, trails, or latrines—may create zones or reduced use by prey, as in the landscape of fear hypothesis (also see Gálvez and Hernandez 2022). This, in turn, may contribute to spatiotemporal variation in the influence of prey species on the vegetation, such as through seed dispersal, seed predation, and herbivory.

In this study, we derived temporal proximity from intervals between location visits recorded by camera traps, which represents an Eulerian alternative to spatial proximity logging with GPS tags. Temporal proximity has been used before to study interspecific interactions between mesopredators (Harmsen et al. 2009; López-Bao et al. 2011; Wang and Fisher 2012), but never to study predator–prey interactions. In addition, using survival analysis with permutations proved to be a valuable statistical method as it allows for the inclusion of censored and nontarget events that occur frequently in camera-trapping data from a field situation. Including, rather than simply omitting, these nontarget observations is crucial as they could possibly influence the target events.

We see four clear advantages of our approach compared to spatial proximity derived from GPS data. First, data can be gathered at relatively low cost and effort compared to GPS tracking. Second, a larger number of individuals can be observed with one camera compared to the single animal that information is gathered on using a GPS device. Third, camera traps require no a priori selection of animal species to track, as the entire community of larger terrestrial mammals is recorded. Last, our approach is noninvasive, as animals do not need to be captured and tagged.

We see three limitations to our approach. First, animals can approach study locations while remaining undetected by the camera, which would mean that temporal proximity of the study animals can be greater than suggested by the data. We believe that the bias will still be small in comparison to the GPS approach, as more animals are represented in the camera-trapping data. Second, it is difficult to determine whether a predator is actively tracking a prey animal or is rather just passing by. A possible improvement to our approach is to record the direction in which animals pass the camera, and to evaluate prey tracking by only considering observations in which the predator and prey animal move in the same direction. Finally, we were not able to account for differences in attractiveness of prey animal species to ocelots. Although agouti is their primary prey on the island, it is also the most common one. As such, it is not possible to simply conclude that ocelots are more strongly attracted to agouti than other prey. Running the analysis separately for each prey species, including the passage of other prey species as a competing event resulting in a censored interval, to determine whether effect sizes per prey species are different was not possible, as this resulted in very few observed intervals per prey species.

We present three recommendations for future studies that plan to record temporal proximity with camera traps. First, the cameras should be deployed for as long as and as uninterrupted possible, as this reduces the relative abundance of censored events. Second, it may be useful to log the direction in which animals are moving to determine whether predators are potentially tracking rather than just passing by. Third, this approach should work better with cameras placed on tracks that animals are known to follow, so as to decrease the likelihood of animals passing behind the camera and to increase the likelihood of capturing predators that are known to often follow tracks.

In conclusion, our findings provide empirical evidence for predator avoidance by prey animals in a tropical moist forest, as well as for prey tracking by ocelots, and show that recording temporal proximity with camera traps is a useful alternative for studying predator–prey interactions.

## Supplementary Data

Supplementary data are available at *Journal of Mammalogy* online.

**Supplementary Data SD1.**—A table showing all observations that were removed from the data set. “Skipped” includes double observations (animal sitting still in front of camera, resulting in a false second observation on departure).

**Supplementary Data SD2.**—Flowchart of data preparation and analysis. The basic structure of our data set is simple: It consists of four columns, indicating the location, date, and time at which an animal was observed, and the animal species. This allows us to calculate time intervals between observations and assign these to either prey–predator or predator–prey events (or censored events, e.g., prey–prey or predator–predator). To create null distributions, we shuffle observation dates within each location between animals.

**Supplementary Data SD3.**—Prey tracking by predators on Barro Colorado Island, Panama, when including competing events of prey species in the analysis. (A) Survival probability curves quantify predator–prey (predator avoidance) intervals recorded by camera traps, fitted using a Weibull distribution. The gray lines represent a thousand random distributions, and the red line is the observed distribution. (B) Constant hazard rate derived from the thousand random distributions (gray bars) and the observed distribution (arrow).

**Supplementary Data SD4.**—Predator avoidance by prey (A, B) and prey tracking by predators (C–F) on Barro Colorado Island, Panama, with time intervals limited to 7 days. (A, C, E) Survival probability curves quantify predator–prey (predator avoidance) intervals recorded by camera traps, fitted using a Weibull distribution. The gray lines represent 999 random distributions, and the red line is the observed distribution. (B, D, F) Constant hazard rate derived from the 999 random distributions (gray bars) and the observed distribution (arrow).

**Supplementary Data SD5.**—Predator avoidance by prey (A, B) and prey tracking by predators (C–F) on Barro Colorado Island, Panama, with predators other than ocelot also included as predators. (A, C, E) Survival probability curves quantify predator–prey (predator avoidance) intervals recorded by camera traps, fitted using a Weibull distribution. The gray lines represent 999 random distributions, and the red line is the observed distribution. (B, D, F) Constant hazard rate derived from the 999 random distributions (gray bars) and the observed distribution (arrow).

gyac091_suppl_Supplementary_Data_SD1

gyac091_suppl_Supplementary_Data_SD2

gyac091_suppl_Supplementary_Data_SD3

gyac091_suppl_Supplementary_Data_SD4

gyac091_suppl_Supplementary_Data_SD5

## Data Availability

We intend to archive data and code for the analyses in Dryad. The underlying camera-trapping material is currently stored on the platform Agouti as part of a larger project. We intend to archive the data in GBIF and the images in Zenodo once the project is completed.
