# Supplementary material for: Prey tracking and predator avoidance in a Neotropical moist forest: a camera-trapping approach
Source: J Mammal. 2022 Nov 11;104(1):137–45. doi: 10.1093/jmammal/gyac091 (PMC10107427; doi:10.1093/jmammal/gyac091)
Supplement: gyac091_suppl_Supplementary_Data_SD1 [file gyac091_suppl_supplementary_data_sd1.docx]

| **Class** | **Scientific name** | **Number of observations** |
| --- | --- | --- |
|  |  |  |
| *False observations*  No animal (false triggers and blanks) | - | 2318 |
| Skipped (double entries) | - | 107 |
| Unknown (unidentifiable) | - | 102 |
|  |  |  |
| *Birds* |  |  |
| Crested guan | *Penelope purpurascens* | 71 |
| Small bird unidentified | Aves indet. | 51 |
| Turkey vulture | *Carthartes aura* | 4 |
| Black vulture | *Coragyps atratus* | 3 |
| Common black-hawk | *Buteogallus anthracinus* | 3 |
| Spotted antbird | *Hylophylax naevioides* | 2 |
| Chestnut-backed antbird | *Poliocrania exsul* | 2 |
| Hummingbird | *Trochilidae sp.* | 1 |
| Rufous motmot | *Baryphthengus martii* | 1 |
| White-whiskered puffbird | *Malacoptila panamensis* | 1 |
| Sunbittern | *Eurypyga helias* | 1 |
| Purplish-backed quail dove | *Zentrygon lawrencii* | 1 |
|  |  |  |
| *Mammals* |  |  |
| Baird’s tapir | *Tapirus bairdii* | 27 |
| Mouse unidentified | *-* | 7 |
| Bat unidentified | - | 6 |
| Capybara | *Hydrochoerus hydrochaeris* | 2 |
| Vampire bat | *Desmodontinae sp.* | 1 |
|  |  |  |
| *Reptiles* |  |  |
| Lizard | - | 1 |
|  |  |  |
