# Supplementary figures and images for: Prey tracking and predator avoidance in a Neotropical moist forest: a camera-trapping approach
Source: J Mammal. 2022 Nov 11;104(1):137–45. doi: 10.1093/jmammal/gyac091 (PMC10107427; doi:10.1093/jmammal/gyac091)

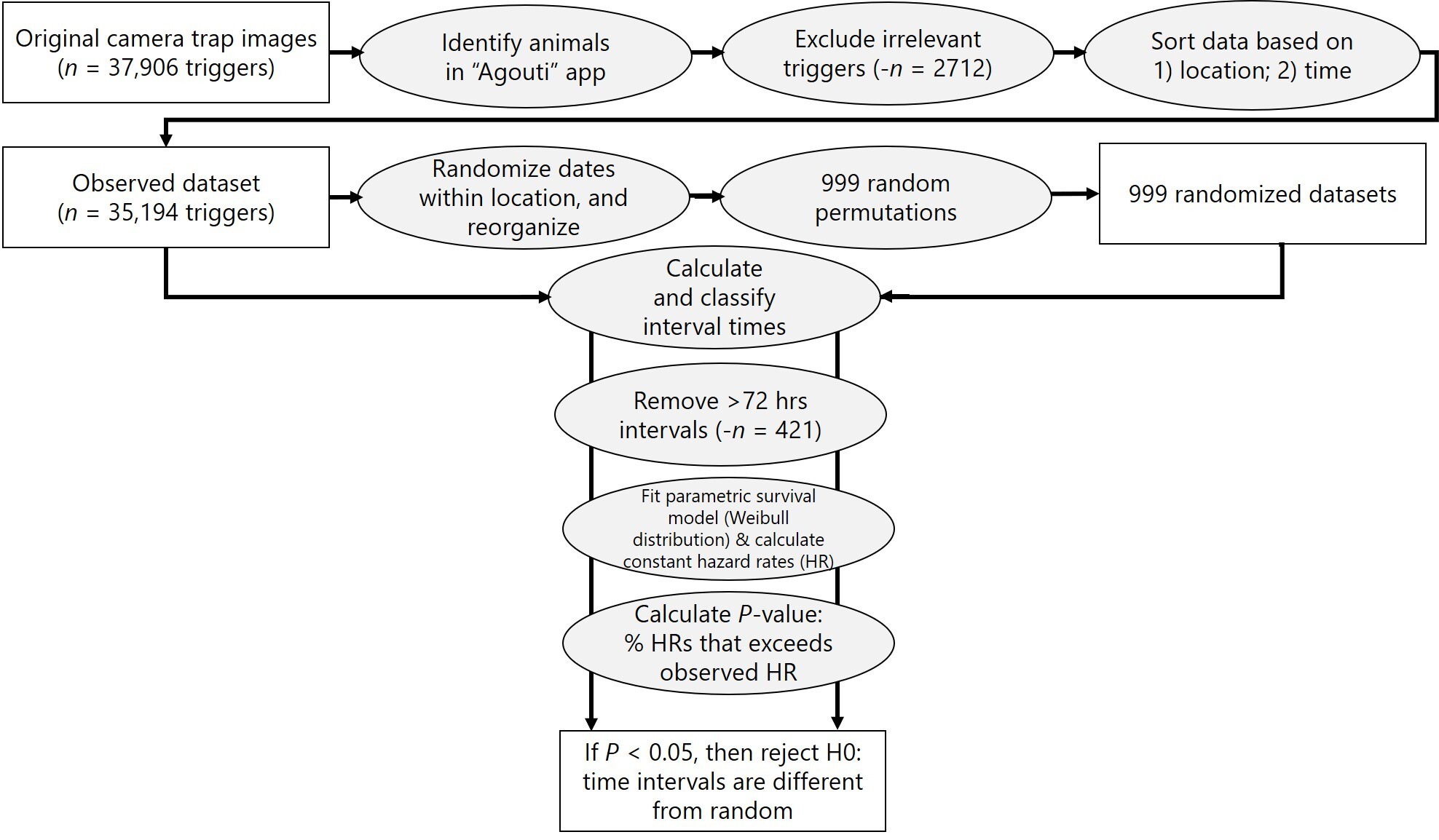

Supplement: gyac091_suppl_Supplementary_Data_SD2 [file gyac091_suppl_supplementary_data_sd2.jpeg]

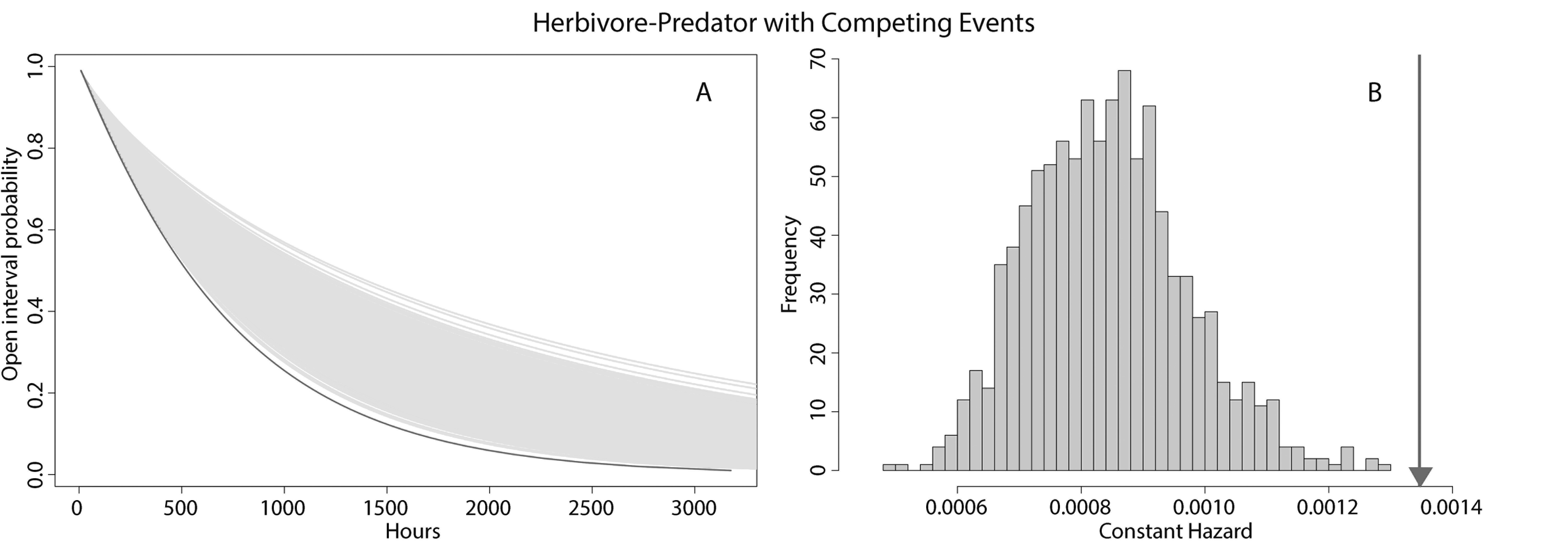

Supplement: gyac091_suppl_Supplementary_Data_SD3 [file gyac091_suppl_supplementary_data_sd3.jpeg]

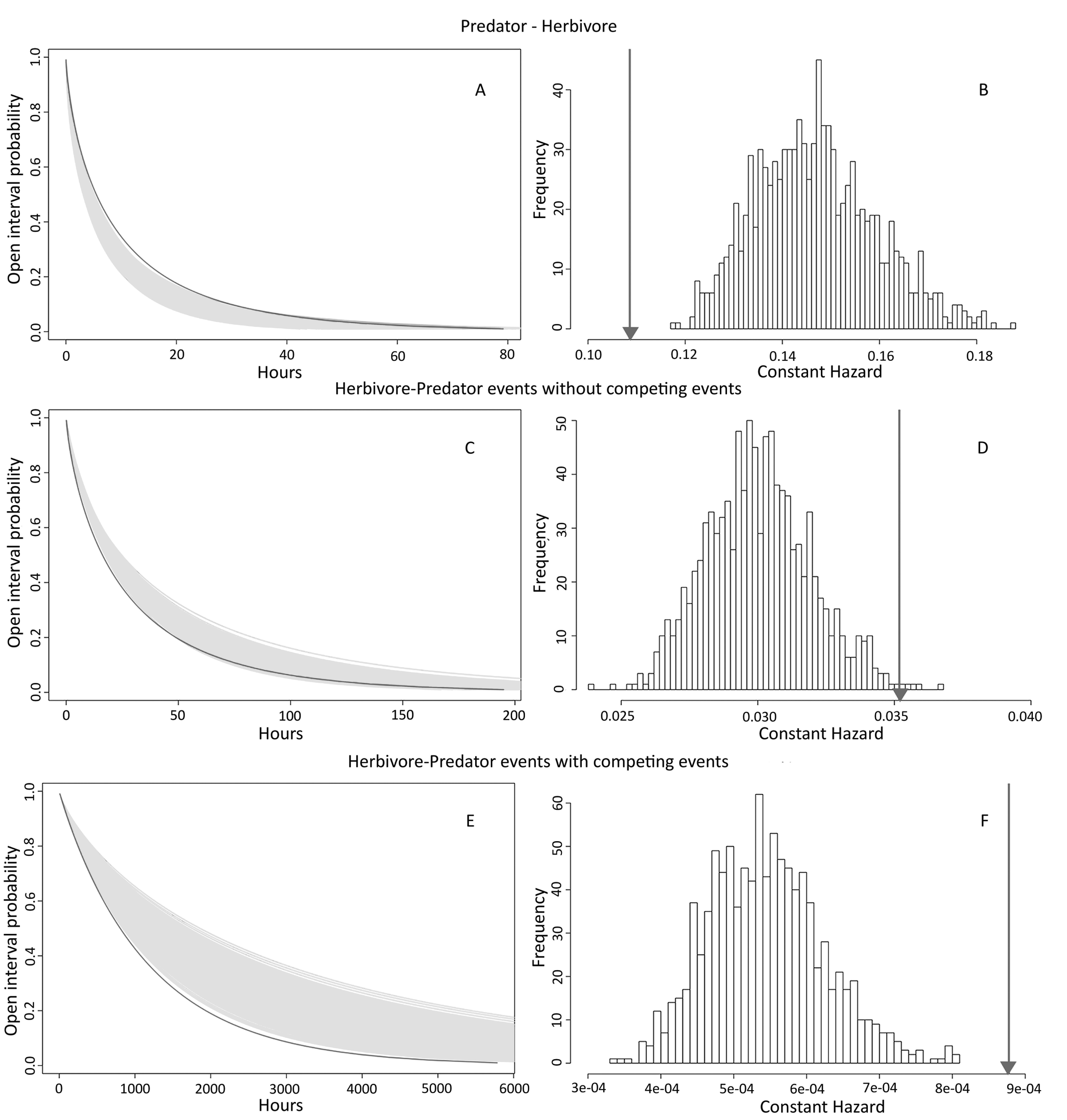

Supplement: gyac091_suppl_Supplementary_Data_SD4 [file gyac091_suppl_supplementary_data_sd4.jpeg]

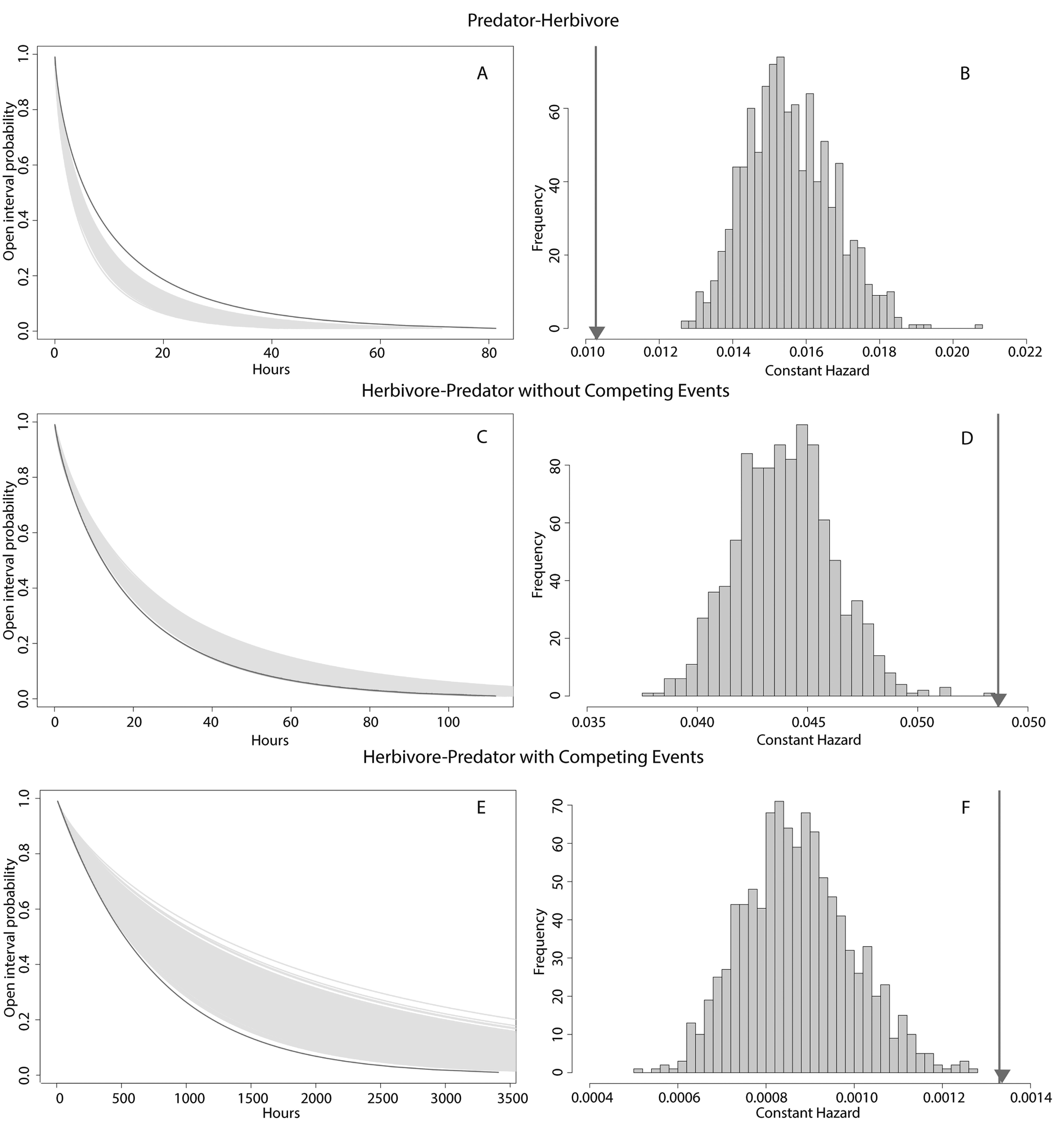

Supplement: gyac091_suppl_Supplementary_Data_SD5 [file gyac091_suppl_supplementary_data_sd5.jpeg]
